# An Active Factor from Tomato Root Exudates Plays an Important Role in Efficient Establishment of Mycorrhizal Symbiosis

**DOI:** 10.1371/journal.pone.0043385

**Published:** 2012-08-21

**Authors:** Shubin Sun, Jingjing Wang, Lingling Zhu, Dehua Liao, Mian Gu, Lixuan Ren, Yoram Kapulnik, Guohua Xu

**Affiliations:** 1 State Key Laboratory of Crop Genetics and Germplasm Enhancement, Nanjing Agricultural University, Nanjing, China; 2 Key Laboratory of Plant Nutrition and Fertilization in Low-Middle Reaches of the Yangtze River, Ministry of Agriculture, Nanjing Agricultural University, Nanjing, China; 3 Department of Agronomy & Natural Resources, ARO, the Volcani Center, Bet Dagan, Israel; National Cancer Institute, United States of America

## Abstract

Root exudates play an important role in the early signal exchange between host plants and arbuscular mycorrhizal fungi. M161, a pre-mycorrhizal infection (*pmi*) mutant of the tomoto (*Solanum lycopersicum*) cultivar Micro-Tom, fails to establish normal arbuscular mycorrhizal symbioses, and produces exudates that are unable to stimulate hyphal growth and branching of *Glomus intraradices*. Here, we report the identification of a purified active factor (AF) that is present in the root exudates of wild-type tomato, but absent in those of M161. A complementation assay using the dual root organ culture system showed that the AF could induce fungal growth and branching at the pre-infection stage and, subsequently, the formation of viable new spores in the M161 background. Since the AF-mediated stimulation of hyphal growth and branching requires the presence of the M161 root, our data suggest that the AF is essential but not sufficient for hyphal growth and branching. We propose that the AF, which remains to be chemically determined, represents a plant signal molecule that plays an important role in the efficient establishment of mycorrhizal symbioses.

## Introduction

Arbuscular mycorrhizal (AM) symbioses occur between most terrestrial plants and fungi of the phylum *Glomeromycota* [1]. The interaction starts with a fine-tuned signal exchange between the two symbiotic partners [2]. Prior to physical contact, the host secretes signal molecules, termed branching factors, that stimulate hyphal growth and branching, while the fungi synthesize and release the so-called Myc factors, which trigger host responses [3], [4], [5], [6], [7]. Despite the recent identification of the major signals used by the host and microsymbionts, the complete spectrum of signaling molecules remains elusive [8].

The identification of the Myc factors used by mycorrhizal fungi to induce host responses involved a tremendous amount of effort [3], [5], [9], [10]. Kosuta *et al*. [11] demonstrated that a diffusible factor secreted by germinating arbuscular mycorrhizal fungi (AMF) spores was able to trigger the expression of a symbiosis-responsive gene (*MtENOD11*) in the host root of *Medicago truncatula* prior to any physical contact. Olah et al. [12] showed that a diffusible factor from AMF was able to stimulate lateral root formation in *Medicago truncatula*. It is unclear whether the *MtENOD11*-inducing and the lateral root-inducing signals are the same molecule. The perception of such diffusible molecules appears to involve the common symbiosis genes in legumes [12]. Navazio *et al.* found that a diffusible molecule constitutively released by the mycorrhizal fungal spores is perceived by the plants via Ca^2+^-mediated signaling [13]. A Myc factor from *G. intraradices*, which turns out to be a lipochitooligosaccharide (Myc-LCO) similar to rhizobia-derived Nod factors, was recently identified [14], [15]. Such Myc factors are thought to be perceived by the same or homologous receptors as Nod factors [14].

AMF sense components of root exudates secreted by the plant [16], [17], [18], [19], [20]. Buee et al. [17] isolated a semi-pure fraction from the exudates of carrot hairy roots that was highly active on the germinating spores of AMF. Tamasloukht et al. [18] found that partially purified exudates induced mitochondrial-related gene expression and fungal respiration during the developmental switch from asymbiosis to pre-symbiosis in AMF. The exudates from two tomato (*Solanum lycopersicum*) mutants, *pmi1* and *pmi2*, that were unable to form AM symbioses, failed to stimulate growth of *G. intraradices* [19], [20]. Together, these results suggest that AMF perceive a signal from the host, *via* root exudates, that stimulates active fungal growth.

Attempts have been made to identify the fungal branching factors. Studies using dialysis membranes indicated that the molecular weights of the branching factors were below 500 Da [21]. Buee et al. [17] proposed that the key plant signal involved in the development of AM fungi is possibly a flavonoid or compound synthesized *via* the flavonoid pathway. The stimulatory effect of the flavonoids is even more pronounced in the presence of CO_2_ [22]. Preliminary characterization of the root exudates that reduced AM fungal proliferation *in vitro* suggests that the methanol-soluble inhibitory moieties are heat labile, bind to polyvinyl polypyrrolidone, and are non-volatile [23]. Strigolactones were chemically identified as hyphal branching factors in the root exudates of AM host plants [24], [25]. Recently, Nagahashi et al. [8] reported that 2-hydroxy fatty acids are another putative category of root exudate signals perceived by *Gigaspora* species that stimulate hyphal branching of germinated AMF spores.

The exudates of M161, a pre-mycorrhizal infection (*pmi*) mutant of micro tomato, are unable to stimulate hyphal growth of *G. intraradices*. Gadkar *et al*. [23] reported that M161 root exudates contain a fraction that inhibits spore germination and hyphal tip growth prior to any physical contact between the host root and fungus. In the present study, we identified the chemical signal(s) involved in the initial stage of the symbiosis by comparing the components of root exudates from wild-type and M161 plants. We found that the M161 exudates lack a methanol-soluble active factor that plays an important role in the growth and branching of *G. intraradices* at the pre-infection stage and, subsequently, in the formation of viable new spores.

## Results

### Wild-type, but not M161, Roots Induce Hyphal Growth and Branching and Generate Viable new *G. intraradices* Spores *in vitro*

**Figure 1:**
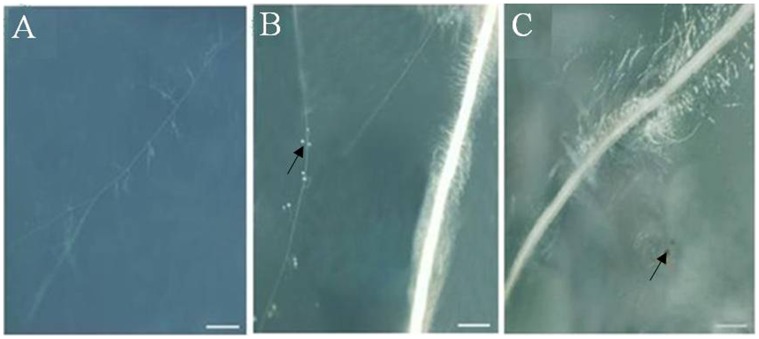
The dual root organ culture system. WT (a,b) and M161 (c) roots cultured *in vitro* with *G. intraradices.* a, Growth and branching of *G. intraradices* spores in the culture with WT roots (bar = 150 µm). b, Generation of progeny spores (arrow) in the WT root culture (bar = 900 µm). c, Abortive *G. intraradices* spores (arrow) in the M161 root culture (bar = 350 µm).

Wild-type (WT) and M161 tomato dual root organ culture (DROC) systems were established using *Agrobacterium rhizogenenes* and AMF, and the ability of these systems to support the *G. intraradices* life cycle was tested. In the WT hairy root culture, *G. intraradices* hyphal growth and branching were observed in the early stages of incubation, and viable progeny spores were subsequently formed (Figure 1a, b). The mean rate of arbuscule colonization of root segments in the later stages of incubation was 7.9% under the low-Pi conditions in the DROC. In contrast, the M161 hairy roots resisted arbuscule colonization (Figure 1c). Neither active mycelia (characterized by growth and branching) nor development of new *G. intraradices* spores was observed at the pre-infection stage in DROC (Figure 1c).

### Identification of a Single Active Factor that Distinguishes WT from M161 Roots

The differences between M161 and WT were evident at early stages of incubation (Figure 1). We hypothesized that the root exudates play a role in this process. To analyze the exudates of WT and M161 hairy roots, lyophilized culture samples were individually dissolved in EtOH and passed through a C18 column, and aliquots were loaded onto a Waters HPLC system and eluted with EtOH. The eluted fraction was used for HPLC analysis.

**Figure 2:**
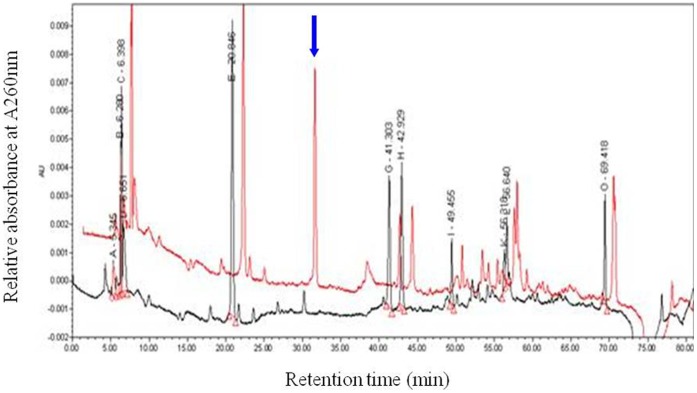
Separation of WT and M161 exudates on an HPLC system. Red and black represent spectra of the WT and M161 exudates, respectively. Aliquots were loaded onto an HPLC column and eluted at 0.8 ml/min with a linear gradient from 1∶99 (v/v) methanol: 1% acetic acid to 99∶1 for 80 min. The arrow indicates the single differential peak (at 30 min) between the WT and M161 exudates.

HPLC chromatograms (at A260 nm) revealed the existence of a single distinct peak at a retention time of 30 min in WT but not M161 mutant exudates (Figure 2). This chemical component(s) corresponding to the differential peak was prepared using a semi-preparative column, and was named the active factor (AF). As a negative control, component(s) of the peak with a retention time of 20 min, which were present in both M161 and WT exudates, were simultaneously prepared and named the non-active factor (NAF).

### The AF Stimulated Hyphal Growth in the Presence of M161 Roots

**Figure 3:**
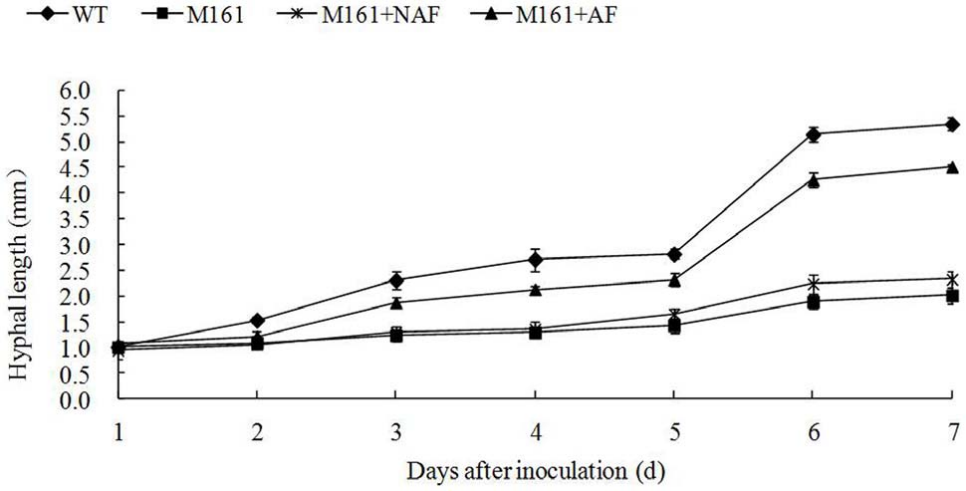
The AF promotes *G. intraradices* hyphal growth in the DROC. The hyphal length of each *G. intraradices* spore was measured each day for 1 week after adding the AF to the DROC. The cultures containing WT and M161 roots without any additive served as the positive and negative control, respectively. The cultures containing M161 and NAF served as a second negative control. Error bars indicate SE (*n* = *3*) of three biological replicates.

To analyze the role of the AF in the AMF life cycle, we added the isolated AF to the DROC medium containing M161 roots and evaluated the response of *G. intraradices* to these conditions. The AMF hyphae exhibited vigorous growth and branching after 2 days of incubation. The AF continued to stimulate hyphal growth and branching throughout the experiment. Total hyphal length was recorded 1 week after the initial stimulation (Figure 3). The AF increased hyphal length 2.2-fold at 5 d and 4.2-fold at 7 d relative to the length at 1 d, compared with 1.4-fold and 2.0-fold in the control without AF or 1.7-fold and 2.4-fold in the control with the NAF, respectively (Figure 3). To determine if the AF is a known branching factor(s), GR24, a synthetic analogue of strigolactone, was used to perform the complementation assay. GR24 (10^−7^ M) resulted in increased hyphal length at 4–10 d compared with the control, but the extent of the increase was much less than in the presence of the AF (Figure S1, Figure 3).

### The AF Induced the Formation of Mycorrhizae in the Presence of M161 Roots

**Figure 4:**
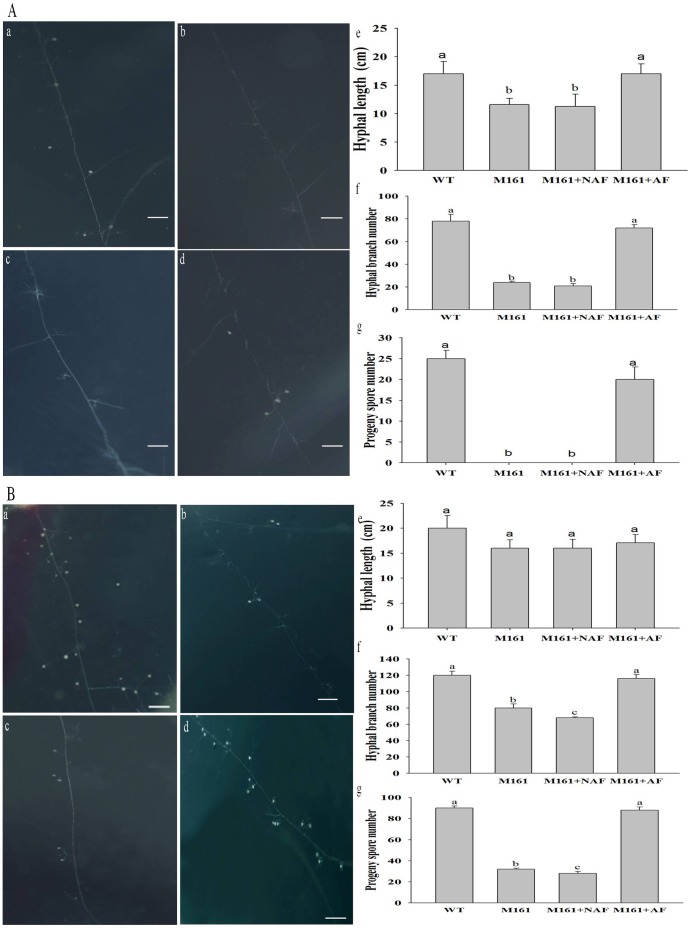
The effect of the AF on hyphal growth and branching and the generation of new spores of *G. intraradices.* Four weeks (A) and six weeks (B) after inoculation with *G. intraradices.* The DROCs containing the AF (A,d; B,d) and DROCs lacking the AF (A,b; B,b) were used as the positive and negative control, respectively. The DROCs containing NAF (A,c; B,c) were used as another negative control. The length of the hyphae protruding from each spore was measured with a micrometer, and the number of hyphal branches was determined (A,e,f; B, e,f). The number of the generating new sp1ores was counted (A,g; B,g).

Four weeks after inoculation with *G. intraradices* spores in the DROCs, the extraradical mycelium was still growing and hyphal branching was significantly greater than in the two negative controls (Figure 4A: b, c, d, e, and f). At this stage, newly formed spores were also present in the culture treated with the AF (Figure 4A: d and g), which is an indication of successful mycorrhizal establishment from the fungal viewpoint. Meanwhile, M161 roots grown in the absence of the AF showed a resistant phenotype. Few active mycelia and no new spores emerged from the inoculum area of *G. intraradices* (Figure 4A: b, c, e, f, and g).

At 35 d after inoculation, more mycelia and new spores had emerged in the DROCs lacking the AF, and the number of new spores increased further 42 d after inoculation (Figure 4B: b, c, e, f, and g). However, there was a large difference in the susceptibility of the M161 roots to mycorrhizal colonization in the presence and absence of the AF (Figure 4B: b, d, f, and g). The sporulation rate in the presence and absence of the AF was 8098±152 (*n* = 5) and 756±106 per Petri dish (*n* = 5) at 47 d after inoculation, respectively.

### The Stimulatory Effect of the AF on Hyphal Development of *G. intraradices* Requires M161 Roots

**Figure 5:**
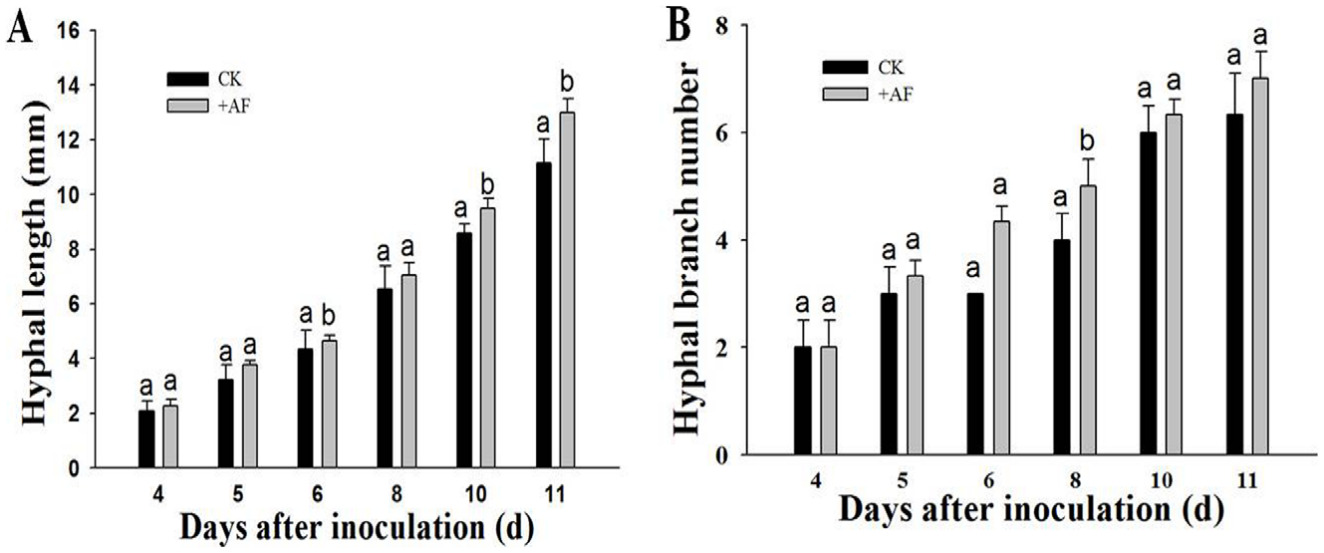
The effect of the AF on hyphal growth and branching of *G. intraradices* in the absence of M161 roots. The surfaces of the spores of *G. intraradices* were sterilized and placed on M solid medium without the roots of M161. The final concentration of the AF was 6.5 µg/mL (marked as +AF). CK represents the negative control without any additive. Hyphal length (a) and the number of hyphal branches (b) were measured for each spore. Error bars indicate SE (*n* = *3*) of three biological replicates.

To determine if the AF is sufficient for mycorrhization, we investigated the effect of the AF on hyphal development of *G. intraradices* grown aseptically in the absence of M161 roots. The results showed that the AF stimulated hyphal growth and branching, but the increase degree was not significantly comparison with that of the control (Figure 5a,b). These results indicated that the AF affected the development of *G. intraradices via* activation of the plant-mycorrhizal fungus interaction.

## Discussion

Identification of the pre-mycorrhizal infection mutants of host plants (myc^−^) provides excellent starting materials for categorizing the complex steps that occur during the establishment of the symbiosis. The *pmi* tomato mutant, M161, which reduces spore germination and hyphal proliferation of AMF during the early stages of infection, provides the opportunity to examine the molecular signal during the early stages of infection. In addition, roots in the dual root organ culture (DROC) system, which was used to analyze the biochemical, genetic, and physiological relationships between AMF and their hosts, showed similar mycorrhizal characteristics as the plants from which they developed [26]. In the present work, we used M161 to study the mechanism(s) involved in the communication between the symbionts at the early stage of the symbioses using DROCs.

The presence of branching factors in the root exudates of AM host plants provides direct evidence for signaling prior to physical contact between the plant and fungal symbionts [7], [9]. We isolated an AF that was present in WT but not M161 root exudates, and demonstrated its ability to stimulate spore germination and hyphal growth of *G. intraradices in vitro*. We found that the AF rescued the inability of the M161 mutant to form normal AM symbioses at the pre-mycorrhizal infection stage, and even at a later stage. The growth of extraradical mycelium and the production of new spores after the addition of the AF in the M161 DROC were almost the same in the WT DROC(Figure 4). Gadka et al. [23] showed that the exudates of the M161 mutant contain a fraction that inhibits hyphal tip growth of *G. intraradices.* However, in this study, we present evidence that the M161 exudates lacked an active factor that stimulated hyphal growth, branching, and new spore production of *G. intraradices* in DROC. We further examined whether the AF is sufficient or not for fungal development. The significant difference of the hyphal growth of *G. intraradices* in the presence and absence of the M161 roots (Figure 3, 5) indicates that the AF requires some active substances in the roots of M161 to stimulate growth and branching. Thus, the AF is necessary but not sufficient for the efficient establishment of a mycorrhizal symbiosis.

The chemical components of the branching factor have remained elusive partially due to the extremely low concentration of metabolites produced and their relative instability [24]. The AF isolated in this work seems to have specific properties. After C18 fractionation, the lyophilized exudates of WT and M161 plants were each resolved in methanol, and then eluted with a linear gradient of methanol:1% acetic acid in an HPLC system. Therefore, we propose that the AF is a methanol-soluble compound. To determine if the AF belongs to the category of strigolactones that was described to play an important role in presymbiosis, we compared the AF to GR24, a synthetic analogue of strigolactone, using HPLC analysis. We found that the AF was not GR24 (data not shown). Together with the results presented in Figure S1, we conclude that the AF that plays an important role during the preinfection stage with M161 is not this type of strigolactone (GR24).

In conclusion, we purified an AF from the exudates of WT roots, and obtained striking evidence that the AF significantly stimulates the growth of the extraradical mycelium at the preinfection stages, and then the production of new spores in DROC with M161. AF appears to be a key component in the molecular dialogue associated with symbiotic infection. However, we were unable to characterize this chemical compound due to its extremely low concentration. A detailed characterization of the AF molecule will reveal why M161 is incapable of forming normal mycorrhizae and further our understanding of the molecular mechanism underlying the formation of mycorrhiza between the host plant roots and the AMF.

## Materials and Methods

### Establishment of the *in vitro* Dual Root Organ Culture (DROC) System

The seeds of wild-type (WT) tomato (*Lycopersicon esculentum* L. cv. Micro-Tom) and its mycorrhizal defective mutant M161 were kindly provided by Dr. Yoram Kapulnik. Roots were cultured according to the protocols described by Becard and Piche [27] and Gadkar et al. [23], using M medium solidified with 0.4% Phytagel (Sigma). Phosphorus was routinely omitted, as the gelling agent contributed a significant amount (about 0.1% by weight) of this element [28]. The *G. intraradices* inoculum provided by Dr. Yoram Kapulnik consisted of cubes of medium containing the spores, hyphae, and root fragments [23]. The dual root organ culture system is referred to as DROC in the text. The arbuscular colonization rate of *G. intraradices* in the agar culture was counted as described previously [29].

### Collection of Root Exudates

To collect exudates, WT and M161 roots were grown on solid M medium. The plates were incubated in the dark at 28°C for about 10 days, until new lateral roots had formed on each of the roots and the culture was healthy and white. Then, the roots were gently lifted from the medium and transferred to an Erlenmeyer flask (500 ml) containing 100 ml of liquid M medium. These flasks were incubated on a rotary shaker set to give gentle agitation (80–100 rpm) in the dark. The medium was replaced with fresh medium under sterile hood conditions every 5 days, and the culture was maintained under these conditions for 2 to 3 weeks. The medium, which accumulated root exudates during the incubation, was transferred to a glass beaker and lyophilized. The dry powder was stored in desiccators in the dark at −20°C.

### Purification of the Exudates and Preparation of the Active and Non-active Factors

Lyophilized root exudates (WT and M161) were dissolved in 2 ml of 50% EtOH, mixed well, and centrifuged. The root exudates were loaded onto C18 columns, and salts and vitamins were washed from the root exudates with 60% EtOH. The eluted fraction was then injected into an HPLC system (Venusil MP, Agela Technologies) fitted with a C18 column (4.6×250 mm) and the sample was eluted at a rate of 0.8 ml/min with a linear gradient from 1∶99 (v/v) methanol:1% acetic acid to 99∶1. The differential peak (active factor, AF) and another peak (non-active factor, NAF) were prepared using an Econosil C18 semi-preparative column (250×10 mm), as described in Volpin *et al*. [30], respectively.

### The AF Complementation Bioassay

*G. intraradices* spores were used to study the effects of the AF and NAF on hyphal growth and branching. The spores were surface-sterilized and placed on solid M medium. AF and NAF were added to DROC at a final concentration of 6.5 µg/mL, respectively. The length of the hypha of each spore was measured with a micrometer and the total number of hyphal branches was determined as described by Gadkar et al. [23].

To determine the effect of the AF on hyphal growth and branching of *G. intraradices* in the absence of M161 roots, the AF was added to Petri dishes without any roots at a final concentration of 6.5 µg/mL.

## Supporting Information

Figure S1 **The effect of different concentrations of GR24 on** ***G. intraradices*** **hyphal length.** CK represents a control without the AF.
(TIF)
