# Supplementary figures and images for: An Active Factor from Tomato Root Exudates Plays an Important Role in Efficient Establishment of Mycorrhizal Symbiosis
Source: PLoS One. 2012 Aug 21;7(8):e43385. doi: 10.1371/journal.pone.0043385 (PMC3424123; doi:10.1371/journal.pone.0043385)

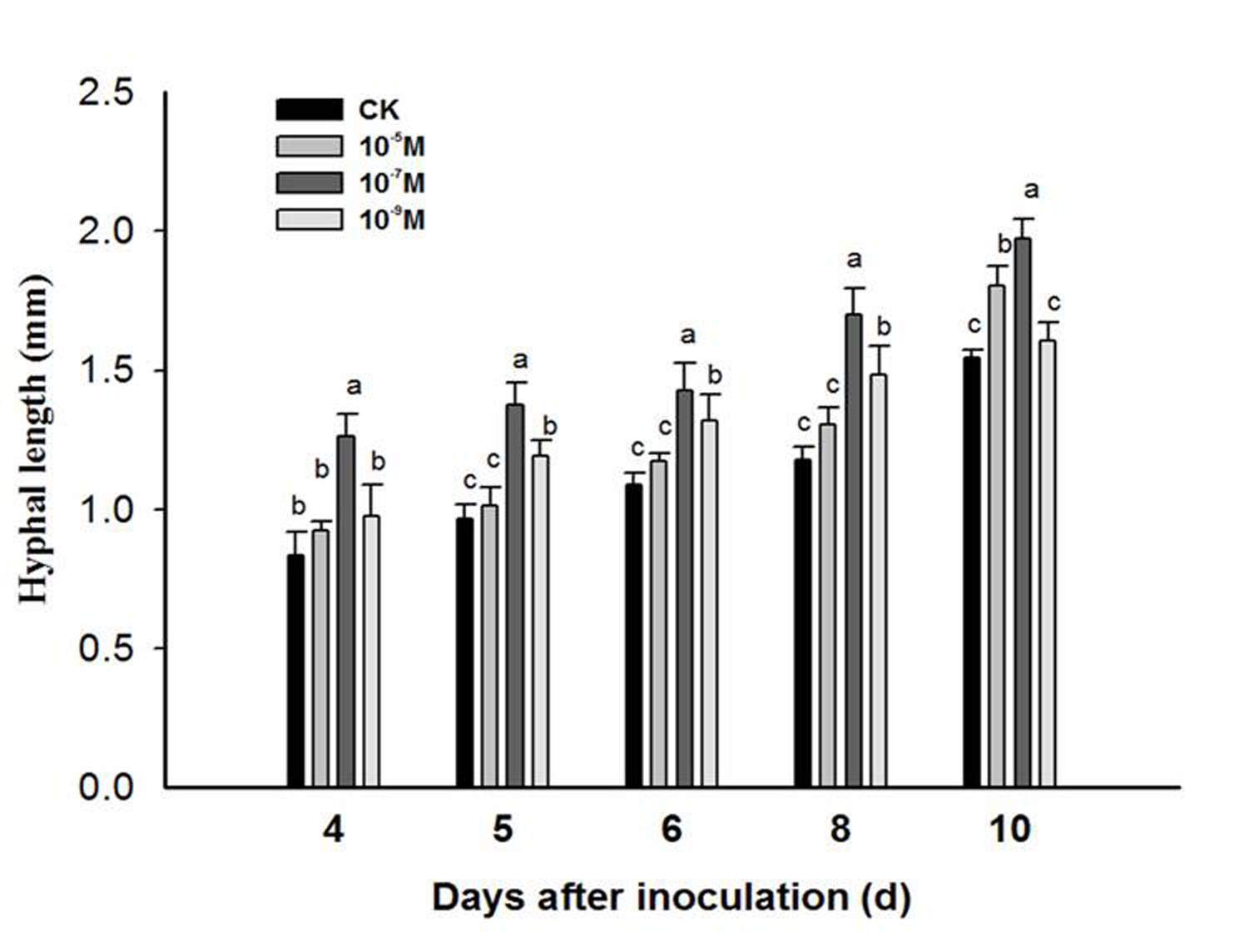

Supplement: Figure S1 — The effect of different concentrations of GR24 on G. intraradices hyphal length. CK represents a control without the AF. (TIF) [file pone.0043385.s001.tif]
